# Cooperative Stimulation of Dendritic Cells by *Cryptococcus neoformans* Mannoproteins and CpG Oligodeoxynucleotides

**DOI:** 10.1371/journal.pone.0002046

**Published:** 2008-04-30

**Authors:** Jennifer M. Dan, Jennifer P. Wang, Chrono K. Lee, Stuart M. Levitz

**Affiliations:** 1 Department of Microbiology and Immunology Training Program, Boston University School of Medicine, Boston, Massachusetts, United States of America; 2 Division of Infectious Diseases, Department of Medicine, University of Massachusetts Medical School, Worcester, Massachusetts, United States of America; Pasteur Institute, France

## Abstract

While mannosylation targets antigens to mannose receptors on dendritic cells (DC), the resultant immune response is suboptimal. We hypothesized that the addition of toll-like receptor (TLR) ligands would enhance the DC response to mannosylated antigens. *Cryptococcus neoformans* mannoproteins (MP) synergized with CpG-containing oligodeoxynucleotides to stimulate enhanced production of proinflammatory cytokines and chemokines from murine conventional and plasmacytoid DC. Synergistic stimulation required the interaction of mannose residues on MP with the macrophage mannose receptor (MR), CD206. Moreover, synergy with MP was observed with other TLR ligands, including tripalmitoylated lipopeptide (Pam3CSK4), polyinosine-polycytidylic acid (pI:C), and imiquimod. Finally, CpG enhanced MP-specific MHC II-restricted CD4^+^ T-cell responses by a mechanism dependent upon DC expression of CD206 and TLR9. These data suggest a rationale for vaccination strategies that combine mannosylated antigens with TLR ligands and imply that immune responses to naturally mannosylated antigens on pathogens may be greatly augmented if TLR and MR are cooperatively stimulated.

## Introduction

Pattern recognition receptors (PRRs) recognize evolutionarily conserved molecular motifs on pathogens. PRRs are employed by the host immune system to evoke an immediate response to the invading pathogen through the production of inflammatory cytokines and chemokines. Because of the efficiency of this response, PRRs are attractive candidates as adjuvants for vaccines. PRRs span a broad range of receptors including Toll-like receptors (TLRs), carbohydrate-binding calcium-dependent type lectin receptors (CLRs), nucleotide binding oligomerization domain like receptors and intracellular viral receptors [1].

The 11 human TLRs and 13 mouse TLRs [2] are membrane-bound receptors which recognize pathogen-associated molecular patterns (PAMPs). TLRs can be either expressed on the cell surface (e.g., TLRs 1,2,4) or on endosomal vesicles (TLRs 3, 7-9). One particular TLR agonist undergoing clinical trials is synthetic cytosine phosphate guanine (CpG)-containing oligodeoxynucleotides (ODN) [3]. CpG is taken up by cells in a clathrin-dependent manner and binds to TLR9 which is recruited from the endoplasmic reticulum to the endosome [4]. This triggers the cytoplasmic Toll-IL-1R domain of TLR9 to bind to the adapter molecule, myeloid differentiation marker 88 (MyD88) [5]. MyD88 diverges into two signaling pathways, one mediated by NF-kB activation and one mediated through interferon regulatory factor (IRF) 7 activation [6].

CLRs recognize particular carbohydrate residues via their extracellular carbohydrate recognition domain. CLRs function both in leukocyte trafficking and antigen recognition [7]. Distinct cytoplasmic motifs on CLRs mediate signaling and trafficking into vesicles. For example, the macrophage mannose receptor (MR, CD206), traffics to early endosomes while the dendritic cell ICAM3-grabbing non-integrin (DC-SIGN, CD209) traffics to late endosomes and early lysosomes [8]. MR preferentially recognizes terminal mannose residues while DC-SIGN recognizes internal mannose residues [7].

The opportunistic fungal pathogen, *Cryptococcus neoformans*, is a major cause of morbidity and mortality in patients with impaired CD4^+^ T-cell function, particularly those with AIDS [9]. A family of antigens, termed mannoproteins (MP), has been identified as the immunodominant antigens which stimulate T-cell responses to *C. neoformans* in patients recovered from cryptococcosis and in experimentally infected mice [10]. MP have extensive N-and O-linked mannosylation, which serve as ligands for MR and DC-SIGN on DCs [10]–[12]. DCs can internalize, process and present MP in the context of the MHC II to CD4^+^ T-cells [13].

While MP are promising vaccine candidates, mice that were administered MP with Ribi adjuvant system were only partially protected from subsequent challenge with *C. neoformans* [14]. Perhaps due to its capacity for Th1 skewing, immune responses appear to be a bit more vigorous when MP are administered with complete Freund's adjuvant [15]. However, because Freund's adjuvant is too toxic for use in routine vaccines, other Th1-skewing adjuvants are under investigation, including CpG [16]. Indeed, administration of CpG alone has salutary effects in murine models of cryptococcosis [17]–[19]. Given the potential of CpG as an adjuvant in an MP-based vaccine against cryptococcosis, we studied the responses of murine DCs following stimulation with MP alone and in combination with CpG. We found that that the two stimuli acted synergistically to induce proinflammatory cytokines and chemokines and to promote an MHC II-restricted, antigen-specific CD4^+^ T-cell response. Moreover, synergy required the interaction of mannose residues on MP with MR on DC.

## Results

**Figure 1:**
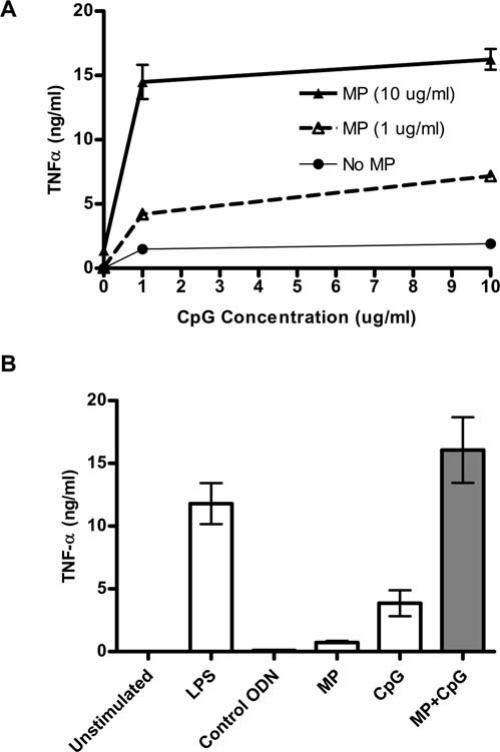
MP synergizes with CpG to stimulate cDCs to produce TNF-α. (A) cDCs were stimulated with CpG ODN 1826 (0, 1, or 10 µg/ml) and/or MP (0, 1, or 10 µg/ml). Supernatants were collected 24 hours later and TNF-α concentrations determined by ELISA. Data are means±SEM of a representative experiment performed in singlicate. A second experiment yielded similar results. (B) MP+CpG synergize to produce more TNF-α than MP or CpG alone. Data represent means±SEM of 6 independent experiments, each performed in singlicate. p<0.001 comparing MP alone or CpG alone with MP+CpG by one way ANOVA with Tukey's multiple comparison test.

Murine cDCs were stimulated with MP, CpG, or MP+CpG and monitored for production of TNF-α. This cytokine plays a critical role in host defenses against cryptococcosis [20]. MP+CpG 1826, a B-type ODN which stimulates murine cDCs, synergized to produce more TNF-α than MP or CpG alone (Figure 1 A). This effect was more than additive, as MP stimulation alone produced little TNF-α. Maximum TNF-α was observed when 10 µg/ml MP was combined with 10 µg/ml CpG. Concentrations of MP higher than 10 µg/ml, when combined with CpG, did not stimulate greater cytokine responses (data not shown). Thus, the dose of 10 µg/ml of MP was used in subsequent cytokine studies on cDCs. The combination of MP+CpG stimulated TNF-α responses comparable to that seen with 1 µg/ml LPS (Figure 1 B). The control ODN 2138 (10 µg/ml), which contains GpC motifs in lieu of CpG motifs, failed to stimulate cytokine release.

**Figure 2:**
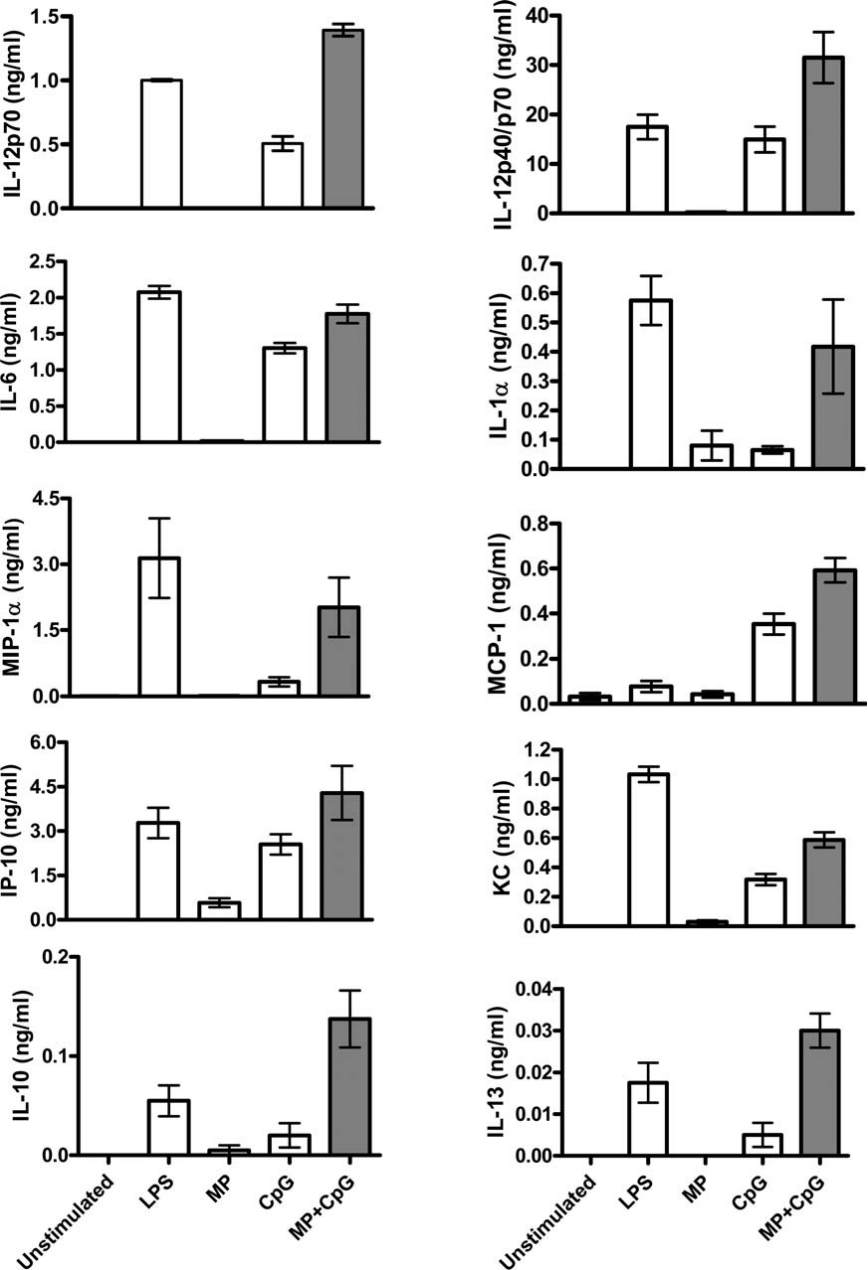
MP synergizes with CpG to stimulate cDC to produce proinflammatory cytokines, chemokines, and immunoregulatory cytokines. cDCs were stimulated as in Figure 1. Data are means±SEM of 4 independent experiments. p<0.001 comparing MP alone with MP+CpG and p<0.05 comparing CpG alone with MP+CpG for IL-6 and IL-12p40/p70. p<0.001 comparing MP alone with MP+CpG and p<0.01 comparing CpG alone with MP+CpG for KC and MCP-1. p<0.05 comparing MP alone or CpG alone with MP+CpG for MIP-1α. p<0.01 comparing MP alone with MP+CpG for IP-10. p<0.01 comparing MP alone or CpG alone with MP+CpG for IL-10. p<0.001 comparing MP alone or CpG alone with MP+CpG for IL-12p70 and IL-13. All statistical comparisons were by one way ANOVA with Tukey's multiple comparison test.

MP+CpG synergized to stimulate cDC production of IL-12p70, as well as enhancing the production of other proinflammatory cytokines, including IL-6, IL-12p40/p70, and IL-1α (Figure 2). Furthermore, MP+CpG augmented the production of the chemokines MIP-1α, KC, MCP-1, and IP-10. However, MP+CpG also enhanced the production of the immunoregulatory cytokines IL-10 and IL-13, although only relatively small amounts of these cytokines were produced. Levels of IL-1β, IL-2, IL-4, IL-5, IL-17, MIG, VEGF, FGF basic, GM-CSF, and IFN-γ were undetectable following stimulation of cDCs with LPS, MP, CpG, and MP+CpG (data not shown).

**Figure 3:**
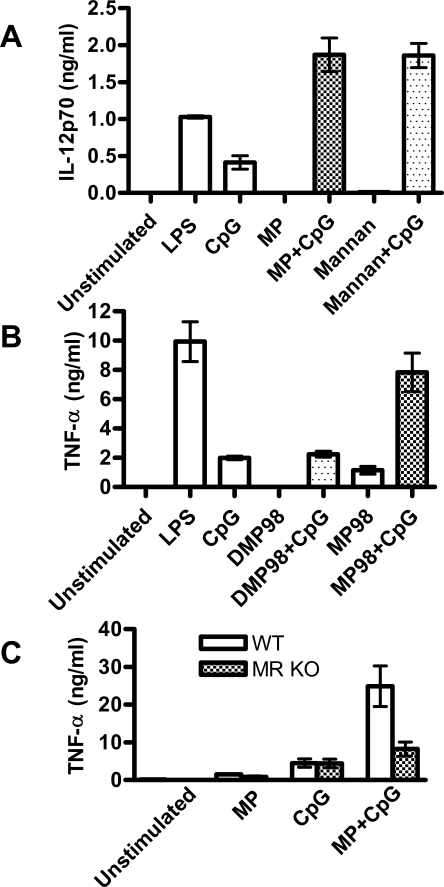
Mannose residues and the MR are critical for synergistic stimulation of cDCs with MP and CpG. (A) cDCs were incubated for 24 hours with 1 µg/ml LPS, Control ODN 2138 (10 µg/ml), CpG ODN 1826 (10 µg/ml), mannan (1 mg/ml), mannan (1 mg/ml)+CpG (10 µg/ml), MP (10 µg/ml), or MP (10 µg/ml)+CpG (10 µg/ml). Supernatants were analyzed by ELISA for IL-12p70. p<0.001 comparing mannan alone or CpG alone with mannan+CpG. p<0.001 comparing MP alone or CpG alone with MP+CpG. (B) Same as figure 3a, except additional stimuli were recombinant MP98 derived from *Pichia pastoris* which was either deglycosylated (DMP98, 1 µg/ml) or left fully mannosylated (MP98, 1 µg/ml). Data are from 3 independent experiments, each performed in singlicate. p<0.001 comparing MP98 alone or CpG alone with MP98+CpG. (C) cDCs were obtained from WT and MR KO mice. Cells were incubated for 24 hours with 10 µg/ml MP, 10 µg/ml CpG, or MP+CpG following which supernatants were analyzed by ELISA. Results are means±SEM of 3 independent experiments each performed in singlicate. p<0.001 comparing MP alone or CpG alone with MP+CpG in WT cDCs. p>0.05 comparing CpG alone with MP+CpG in MR KO cDCs. All statistical comparisons were by one way ANOVA with Tukey's multiple comparison test.

MP consists of a protein core with extensive mannosylation. We hypothesized that synergy of MP and CpG requires mannosylation. To test this hypothesis, we determined whether *Saccharomyces cerevisiae* mannan, which consists of branched mannose chains, could be substituted for MP in mediating synergy with CpG. We found that this was indeed the case; mannan and MP each synergized with CpG to boost IL-12p70 production by cDCs (Figure 3 A). To further test the hypothesis, we compared the synergistic stimulatory capacity of recombinant *C. neoformans* MP98, a highly mannosylated MP produced in *Pichia pastoris,* with a version that was chemically deglycosylated (DMP98) [21] . Enhanced TNF-α was seen when the cDCs were stimulated with MP98+CpG, but not DMP98+CpG (Figure 3 B). Together, these data strongly suggest that the mannose residues are necessary for synergy with CpG.

Next, the role of the MR, CD206, was addressed. cDCs from MR KO mice were stimulated with MP, CpG, and MP+CpG. There was a significant decrease in the amount of TNF-α produced in MR KO cDCs stimulated with MP+CpG compared to WT cDCs stimulated with the same amount of antigens (Figure 3 C). This suggests a prominent role for the MR in driving synergy.

**Figure 4:**
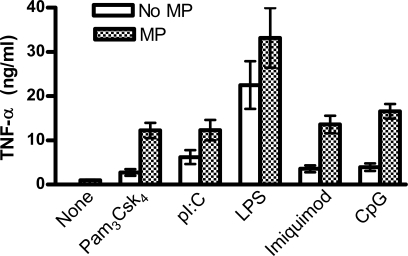
MP synergizes with multiple TLR ligands. cDCs were incubated for 24 hours with 10 µg/ml MP, 10 µg/ml Pam_3_CSK_4_, 10 µg/ml pI:C, 1 µg/ml LPS, 10 µg/ml imiquimod, and 10 ug/ml CpG. Supernatants were collected and analyzed for TNF-α by ELISA. Data represent means±SEM of 4 independent experiments, each of which was performed in singlicate. p<0.001 comparing any TLR ligand alone with the TLR ligand plus MP by the two-tailed paired t-test.

TLR9, the ligand for CpG, utilizes MyD88 as its adapter molecule to signal downstream events. Therefore, as expected, neither CpG nor MP+CpG elicted any TNF-α from cDCs derived from MyD88 KO mice (data not shown). To test whether this synergy was a unique feature of the interaction of MP+CpG, we next tested cDC cytokine release induced by TLR1/2, TLR3, TLR4 and TLR7/8 ligands alone or in combination with MP. Addition of MP resulted in stimulation of significantly greater TNF-α release with all tested TLR ligands (Figure 4). This demonstrates that MP+TLR ligand synergy also occurs following stimulation of TLRs that are located on the cell surface (TLR1/2, TLR4) and that utilize adapter molecules other than MyD88 (TLR3).

**Figure 5:**
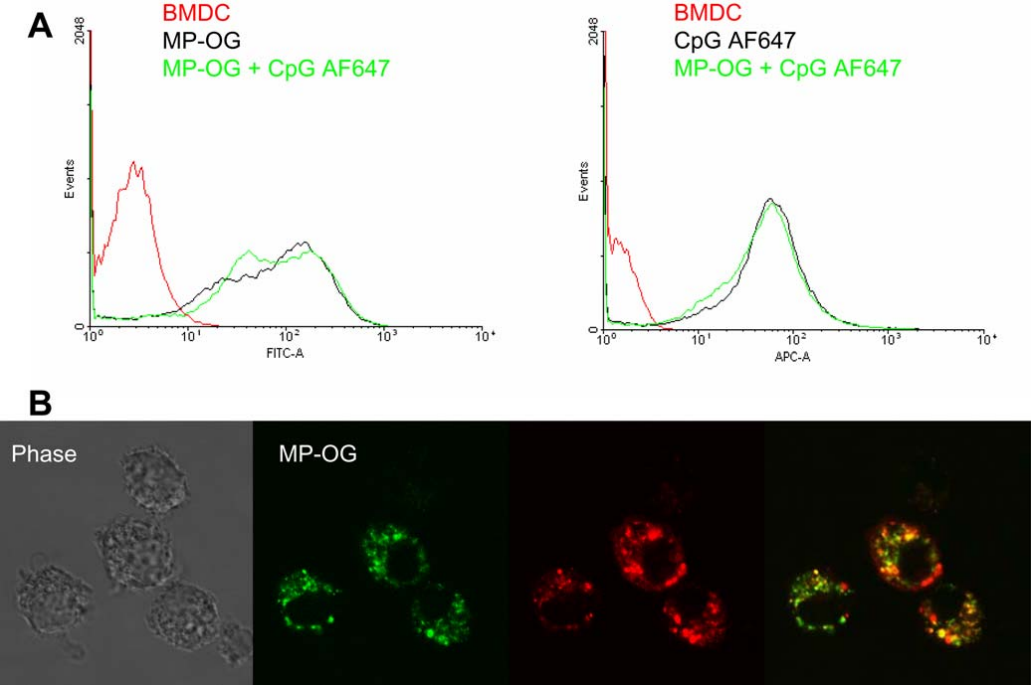
MP and CpG are taken up independently but can colocalize in intracellular compartments. (A) MP-OG (10 µg/ml) and 1 µM CpG ODN 1826-AF647 were incubated with cDCs for 15 minutes and analyzed by flow cytometry. Plots are representative of two independent experiments. The plot on the left demonstrates no effect of CpG AF647 on cellular uptake of MP-OG. The second plot demonstrates no effect of MP-OG on cellular uptake of CpG AF647. (B) cDCs were incubated with 10 µg/ml MP-OG (green) and 1 µM CpG ODN 1826-AF647 (red) for 20 minutes and then analyzed by confocal microscopy. Yellow pseudocolor represents areas of colocalization of MP and CpG within DCs.

To address whether the observed synergy between MP+CpG was due to a possible physicochemical interaction between MP and CpG, WT cDCs were compared for their capacity to take up Oregon Green-labeled MP and Alexa Fluor 647-labeled CpG (Figure 5 A). However, uptake of CpG, as determined by flow cytometry, was similar in the presence and absence of MP. Thus, CpG does not appear to be “carried” by MP into the cell. Nevertheless, by confocal microscopy, although MP and CpG can be found in distinct compartments, some colocalization of CpG and MP inside the cell is also evident (Figure 5 B). These data demonstrating that MP and CpG traffic to similar vesicular structures suggest a potential mechanism whereby the two stimuli may synergize to stimulate cytokine production.

**Figure 6:**
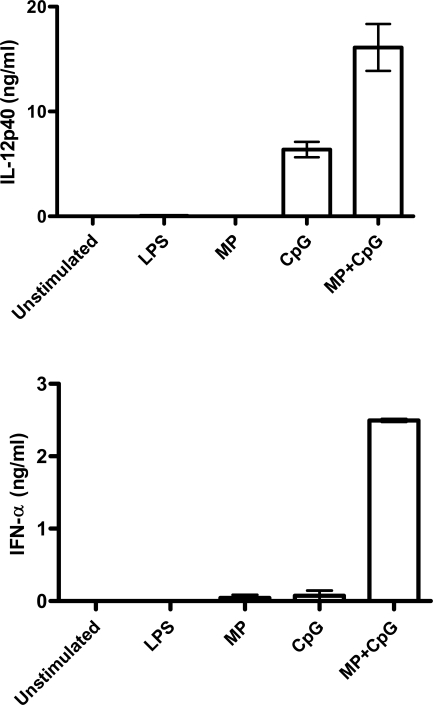
MP+CpG synergize to stimulate murine pDCs to produce IL-12p40 and IFN-α. pDCs were incubated with MP (1 µg/ml), CpG ODN 2336 (1 µg/ml), or MP (1 µg/ml)+CpG (1 µg/ml). Supernatants were collected 40 hours later and analyzed by ELISA. Data are means±SEM of a representative experiment performed in singlicate. Two other experiments yielded similar results. p<0.001 comparing MP alone with MP+CpG and p<0.01 comparing CpG alone with MP+CpG for both IL-12p40 and IFN-α by one way ANOVA with Tukey's multiple comparison test.

The above studies focused on cDCs. As plasmacytoid DCs (pDCs) express high levels of TLR9 [22], we next examined whether MP+CpG would synergize to stimulate murine pDC cytokines. We found that CpG 2336 (1 µg/ml)+MP (1 µg/ml) led to the production of IL-12p40 at levels significantly greater than those induced by CpG (1 µg/ml) alone (Figure 6 A). CpG 2336 is an A-type CpG which stimulates murine pDCs. As pDCs also produce IFN-α in response to the A-type CpG 2336, we measured IFN-α by ELISA and found that MP+CpG stimulated significantly greater amounts of IFN-α from pDCs than CpG alone. (Figure 6 B). Of note, CpG 2336, at 10 µg/ml, induced high amounts of IL-12p40 and IFN-α but the addition of MP did not lead to further increases (data not shown). LPS failed to induce cytokine production, which is consistent with the lack of expression of TLR4 on pDC [22], and thus demonstrates the high purity of the pDC population.

**Figure 7:**
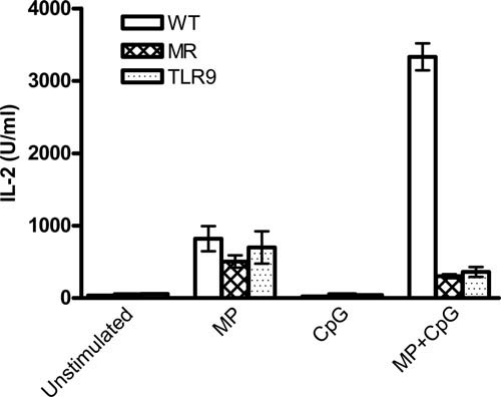
Addition of CpG to MP enhances IL-2 production by an MP specific T-cell hybridoma (P1D6) via a mechanism dependent on MR and TLR9. cDCs were generated from WT, MR KO and TLR9 KO mice and incubated with P1D6 cells in the presence of the indicated stimuli. MP and CpG were both added at 1 µg/ml. Data are means±SEM and are representative of 3 independent experiments with WT and MR KO cDCs and one experiment with TLR9 KO cDCs. p<0.001 comparing MP alone or CpG alone with MP+CpG in WT cDCs by one way ANOVA with Tukey's multiple comparison test.

Finally, we sought to determine whether stimulation of cDCs with MP+CpG would result in an enhanced response of antigen-specific T-cells. For these studies, we utilized the MP-specific T-cell hybridoma P1D6, which produces IL-2 when stimulated by DCs presenting a peptide fragment of MP98 in an MHC II-restricted fashion [12]. In the presence of MP alone, cDCs derived from WT, MR KO, and TLR9 KO mice stimulated similar levels of T-cell IL-2 production. However, stimulation of WT cDCs with MP+CpG resulted in greatly enhanced IL-2 production compared with stimulation with either agent alone (Figure 7). Synergy was not observed with either MR or TLR9 KO cDCs.

## Discussion

In this study, we demonstrate that MP from *C. neoformans* and the TLR9 agonist CpG synergize to stimulate cDCs and pDCs to produce proinflammatory cytokines and chemokines, many of which (e.g., IL-12p70) are associated with induction of Th1-type responses. Moreover, cDCs stimulated with MP+CpG had enhanced IL-2 production by MP-specific CD4^+^ T-cells. Thus, stimulation of the innate immune receptors by mannosylated ligands and CpG has the potential to enhance and bias adaptive immune responses.

We have previously demonstrated that the MR on DC recognizes and internalizes mannose residues on MP [11], [13]. Here these findings are extended to demonstrate that synergy of MP with CpG also is dependent on mannose residues. Synergy was abolished by deglycosylation of MP98 and could be replicated if *S. cerevisiae* mannans were substituted for MP. DCs have multiple receptors that recognize mannose, including DC-SIGN and the SIGNR homologs [23]. However, as synergy was not observed with cDCs from MR KO mice, the MR appears to mediate this effect. Interestingly, in the absence of a TLR ligand, MP stimulated similar amounts of IL-2 production from antigen-specific T-cells co-cultured with either MR KO or WT cDCs. However, when MP+CpG were added, IL-2 synergy occurred only with WT cDCs. Taken together, these data imply that while uptake, processing and presenting of MP can occur independently of the MR, synergistic stimulation of T cells by MP and CpG requires the MR.

Not surprisingly, MP plus CpG synergy in cDCs was also dependent on TLR9 and its adapter protein MyD88, as evidenced by the loss of synergistic TNF-α secretion when cDCs from TLR9 KO or MyD88 KO mice were utilized. However, the TLR9 agonist CpG is not the sole TLR ligand that can synergize with MP to produce proinflammatory cytokines and chemokines. Synergy was also observed with Pam3CSK4 (TLR1/2), pI:C (TLR3), LPS (TLR4), and imiquimod (TLR7/8). This has implications for the design of human vaccines because while human pDCs express TLR9, human cDCs do not [22]. However, both human cDCs and pDCs express other TLRs, including TLR7/8.

We did not observe any appreciable induction of cytokines using MP alone on both cDCs and pDCs. This is in contrast to one study which utilized MP from a different cryptococcal strain [24]. Thus, an adjuvant was necessary for us to produce a proinflammatory milieu. Similarly, we have found that MP does not stimulate upregulation of CD40, CD86, and MHCII on DC (Dan, Kelly, Lee, and Levitz; submitted for publication). The addition of CpG to MP resulted in upregulation of these maturation markers. However, there was no “synergy” as the level of expression of CD40, CD86, and MHCII induced by CpG was the same in the presence or absence of MP (Dan and Levitz, unpublished data).

Other studies have demonstrated cooperative responses between TLRs and other PRRs in the production of cytokines and chemokines by DCs [1]. For example, collaborative induction of inflammatory responses to the fungi *Candida albicans* and *Pneumocystis carinii* has been observed with TLR2 and Dectin-1 [25]–[27]. Similarly, mannans derived from *C. albicans* stimulate phagocytes via both TLR4 and MR [28]. Triggering PRRs on dendritic cells can generate proinflammatory cytokines and chemokines which recruit and activate other effector cells to the area of the invading organism. Ideally, this process then results in killing of the invading organisms by the innate immune response and/or generation of effective adaptive responses [29]. However, it is important to note that MP plus CpG stimulated both proinflammatory and antiinflammatory cytokines, and thus the in vivo effects of cooperative stimulation of the MR and TLRs cannot be predicted with certainty.

It is tempting to speculate that the observed synergy of MP+CpG is dependent upon the triggering of distinct intracellular signals, which then merge to activate NF-kB and IRFs, as has been demonstrated following stimulation of Dectin-1 and TLR2 [30]. MR bears a tyrosine-based sorting motif within its cytoplasmic tail which directs localization of antigen into endosomes [31]. However, whether ligation of MR triggers intracellular signaling cascades is controversial, particularly as there are no known intracellular signaling motifs in the MR [31]. Treatment of macrophages with an anti-MR monoclonal antibody resulted in production of mainly anti-inflammatory cytokines, including IL-10 [32]. However, *Pneumocystis carinii*-induced activation of NF-kB in alveolar macrophages was inhibited by an anti-MR blocking antibody [33]. The MR functions both in pathogen recognition and as an endogenous receptor for secreted proteins [34]. Hence, perhaps it makes teleological sense that stimulation of MRs should not induce a strong proinflammatory response unless a “danger signal” such as a TLR ligand is also present to alert the host to a threatening situation.

The specific intracellular signaling pathways responsible for the observed synergy of MP+CpG remain undefined. Our preliminary studies indicate a role for phosphoinositide 3-kinases (PI3K), as in four independent experiments, the PI3K inhibitors wortmannin and LY294002 each reduced the synergistic production of TNF-α in response to CpG and MP (Dan and Levitz, unpublished data). PI3K have been implicated in signaling events mediated by multiple TLRs, including TLR2, TLR3, TLR4, and TLR9, so they could potentially mediate the observed synergy between MP and other TLR ligands in DCs [35]. Future studies should help clarify this issue.

It has been suggested that DCs “sample” phagosomal and endosomal compartments and that those containing TLR ligands are preferentially processed [36], [37]. As our confocal microscopy studies showed some colocalization of MP and CpG within DC compartments, this may help explain the increased cytokine production and antigen-dependent CD4^+^ T-cell response when DCs were costimulated. However, further studies will be necessary to prove this theory, particularly as a significant amount of internalized MP and CpG localized to distinct compartments. Interestingly, synergistic stimulation of cytokine responses was seen regardless of whether MP was combined with ligands for intracellular or plasma membrane TLRs. However, it remains to be demonstrated whether TLR ligands other than CpG will colocalize with MP in DCs.

The demonstration that TLR ligands synergize with MP has broad implications for the choice of an adjuvant not only for cryptococcal vaccines, but for other antigens with mannose moieties. In particular, one recent study highlighted the efficacy of targeting the MR and utilizing a TLR agonist as an adjuvant for potential chemotherapy in human cancers [38]. The efficiency of the immune response could be greater if the mannosylated antigens and TLR ligands were packaged together so that they are directed to the same compartment in the cell. A similar strategy has been suggested for the design of vaccines containing multiple TLR ligands [36]. It can be predicted though that because the pattern of response varies depending on the individual PRR that is stimulated, that different combinations of PAMPs will elicit distinct responses. While our data lend strong support to the design of vaccines that combine mannosylated antigens with TLR ligands, ultimately the utility of such an approach will require in vivo testing.

## Materials and Methods

### Reagents

MP was prepared as described [14] from culture supernatants of *C. neoformans* acapsular strain Cap 67 (ATCC 52817). The preparation had an endotoxin content of 0.020 EU/μg, as detected by the Limulus amebocyte lysate assay (Associates of Cape Cod, East Falmouth, MA). ODN 1826 (B-type ODN), ODN 2138 (B-type negative control ODN, containing GpC rather than CpG sequences), and ODN 2336 (A-type ODN) were purchased from Coley Pharmaceuticals. Mannan was obtained from Sigma Aldrich. *C. neoformans* MP98 was expressed recombinantly in *P. pastoris* and purified as described [21]. MP98 was deglycosylated (DMP98) by treatment with trifluoromethanesulfonic acid using a commercial kit (GlycoProfile IV chemical deglycosylation kit, Sigma) followed by gel purification, as in previous studies [21]. Deglycosylation was confirmed by loss of periodic acid-Schiff staining and an increase in mobility on SDS-PAGE. Pam_3_CYSK_4_, polyinosine-polycytidylic acid (pI:C), and imiquimod were obtained from Invivogen. LPS from *Escherichia coli* O111:B4 (Sigma) was repurified via a modified phenol re-extraction technique to yield LPS with only TLR4 agonist activity [39].

### Mice

C57BL/6 mice were obtained from Jackson Laboratories. MR (CD206) KO mice were a gift from M.C. Nussenzweig (The Rockefeller University). MyD88 KO and TLR9 KO mice were a gift from S. Akira (Osaka University). All KO mice were backcrossed to a C57BL/6 background for at least ten generations. All animal studies were approved by The University of Massachusetts Medical School's Institutional Animal Care and Use Committee.

### Bone marrow-derived cDCs

Murine bone marrow-derived cDCs were generated following the protocol by Lutz et al. [40], with slight modifications. Briefly, cells obtained from the femurs and tibias of mice were cultured at 2×10^6^ cells per 100×15 mm Petri dishes in 10 mL of R10 media containing RPMI 1640 supplemented with 10% FBS (Tissue Culture Biologicals, Tulare, CA), 100 U/ml penicillin, 100 µg/ml streptomycin, 2 mM L-glutamine, 50 µM 2-mercaptoethanol. R10 media were supplemented with 10% supernatant obtained from the GM-CSF producing J558L cell line at 37° C and 5% CO_2_. Media were replenished on days 3, 6, and 8. On day 9, non-adherent cells were harvested and the cDCs were purified by two passages over an LS column (Miltenyi Biotec) using CD11c^+^ magnetic beads. The cDCs were >90% CD11c^+^ CD11b^+^ B220^−^, as determined by flow cytometry.

### Bone marrow-derived pDCs

To generate pDCs, bone marrow-derived DCs were isolated from femurs and tibias and the RBCs were lysed with RBC lysing buffer (Sigma). Cells were washed 3 times and resuspended in 12 mLs R10 media supplemented with 20 ng/ml recombinant mouse fms-related tyrosine kinase 3 ligand (Flt3L) (R&D). Four mL were added per well of a 6 well plate and the cells were cultured for 7 days without changing the medium. Non-adherent cells were harvested and sorted for CD11c^+^, CD11b^−^, B220^+^ on FACSAria cell sorter (BD Biosystems). Mouse pDC purity was ≥98%.

### Cytokine ELISAs

Purified cDCs (2×10^5^) were incubated with the indicated stimulus for 24 hours in R10 medium containing 20 µg/ml polymyxin B (except for the LPS-stimulated groups) in 48 well non-tissue cultured treated plates. Cell supernatants were collected and analyzed by ELISA using IL-12p70 and TNF-α ELISA kits (eBioscience). Fibroblast growth factor basic, GM-CSF, IFN-γ, IL-1α, IL-1β, IL-2, IL-4, IL-5, IL-6, IL-10, IL-12, IL-13, IL-17, IFN-γ-inducible protein 10, KC, MCP-1, MIG, MIP-1α, TNF-α, and vascular endothelial growth factor were analyzed on a Bio-Plex Luminex-100 station at the Baylor Luminex National Institute of Allergy and Infectious Diseases core facility (Dallas, TX). For pDCs, cells (5×10^4^/well) were incubated with the indicated stimuli for 40 hours in R10 in 96 well flat bottom plates. Supernatants were collected and analyzed by ELISA for murine IL-12p40 (BD Bioscience) and IFN-α (PBL Biomedical Laboratories).

### Flow Cytometry and Confocal Microscopy

MP was labeled with Oregon green in sodium bicarbonate buffer (pH 8.0) following the manufacturer's protocol (Molecular Probes). Excess Oregon green was removed using a G10 Sephadex column (Sigma Aldrich). MP-OG was then dialyzed overnight against water. Alexa Fluor 647 labeled CpG ODN 1826 was obtained from Integrated DNA Technologies and dissolved in DNase-, RNase-free distilled water (Invitrogen). Flow cytometric data were acquired using an LSR Flow Cytometer from BD Biosciences and analyzed using WinMDI software (Scripps Research Institute). Confocal microscopy was performed on a Leica SP2 AOBS confocal microscope (Leica Microsystems) using 35 mm glass bottom dishes (MatTek).

### Stimulation of an MP-specific T cell Hybridoma

CD11c^+^ purified cDCs (1×10^4^/well) derived from WT, MR KO, and TLR9 KO were co-cultured with the MP-specific T-cell hybridoma, P1D6 (1×10^5^/well) for 24 hours in the presence of no stimulus, MP, CpG or MP+CpG [12]. Supernatants were collected and assayed for IL-2 by bioassay using the IL-2-dependent CTLL-2 cell line.

### Statistics

GraphPad Prism Software was used for statistical analyses. When comparing three or more groups, a one way ANOVA with a Tukey multiple correction test was performed. For the experiments in figure 4 comparing TLR agonists in the presence and absence of MP, a two-tailed paired Student t-test with a Bonferroni correction was performed. Significance was defined as p<0.05.
